# Front-Line Emergency Department Clinician Acceptability and Use of a Prototype Real-Time Cloud-Based Influenza Surveillance System

**DOI:** 10.3389/fpubh.2021.740258

**Published:** 2021-11-04

**Authors:** Richard E. Rothman, Yu-Hsiang Hsieh, Anna DuVal, David A. Talan, Gregory J. Moran, Anusha Krishnadasan, Katy Shaw-Saliba, Andrea F. Dugas

**Affiliations:** ^1^Department of Emergency Medicine, Johns Hopkins University, Baltimore, MD, United States; ^2^Ronald Reagan University of California, Los Angeles (UCLA) Medical Center, Los Angeles, CA, United States; ^3^University of California, Olive-View Medical Center, Los Angeles, CA, United States

**Keywords:** influenza, surveillance, emergency department, dashboard, provider acceptability, cloud-based, survey

## Abstract

**Objectives:** To assess emergency department (ED) clinicians' perceptions of a novel real-time influenza surveillance system using a pre- and post-implementation structured survey.

**Methods:** We created and implemented a laboratory-based real-time influenza surveillance system at two EDs at the beginning of the 2013-2014 influenza season. Patients with acute respiratory illness were tested for influenza using rapid PCR-based Cepheid Xpert Flu assay. Results were instantaneously uploaded to a cloud-based data aggregation system made available to clinicians *via* a web-based dashboard. Clinicians received bimonthly email updates summating year-to-date results. Clinicians were surveyed prior to, and after the influenza season, to assess their views regarding acceptability and utility of the surveillance system data which were shared via dashboard and email updates.

**Results:** The pre-implementation survey revealed that the majority (82%) of the 151 ED clinicians responded that they “sporadically” or “don't,” actively seek influenza-related information during the season. However, most (75%) reported that they would find additional information regarding influenza prevalence useful. Following implementation, there was an overall increase in the frequency of clinician self-reporting increased access to surveillance information from 50 to 63%, with the majority (75%) indicating that the surveillance emails impacted their general awareness of influenza. Clinicians reported that the additional real-time surveillance data impacted their testing (65%) and treatment (51%) practices.

**Conclusions:** The majority of ED clinicians found surveillance data useful and indicated the additional information impacted their clinical practice. Accurate and timely surveillance information, distributed in a provider-friendly format could impact ED clinician management of patients with suspected influenza.

## Introduction

Seasonal and pandemic influenza result in up to 960,000 hospitalizations and 80,000 deaths annually in the U.S. (1). Emergency departments (EDs) are one of the most frequent points of entry for initial diagnosis and management of patients with suspected influenza (2), serving as key sentinel surveillance sites (3, 4). Traditional approaches for gathering and distributing surveillance information have relied on collection of data from sentinel sites including EDs, then collating and sharing that information *via* local, regional or national data systems. While broadly useful for public health purposes, intrinsic limitations of traditional influenza surveillance include time lags and loss of local data granularity (5, 6). In addition, communication and delivery methods of sharing surveillance information from local, regional or national public health agencies with front-line clinicians have been challenging, especially during public health emergencies. Front-line clinicians have expressed that surveillance information should be a simple, easily-recognized, localized, authoritative, and practice-focus (7). Eliminating gaps might add value for front-line ED clinicians, with potential utility to inform clinical decisions in the face of seasonal or pandemic influenza.

Several studies from non-ED settings suggest real-time infectious disease surveillance may provide timely actionable information (4, 8–11). Accordingly, advances have been made in cloud-based reporting tools which could complement conventional surveillance systems but add closer to real-time or real-time capabilities (6, 12). Santillana et al. demonstrated ability to track real-time U.S. regional influenza activity using near real-time extracted electronic health record data via cloud-based services coupled with a machine learning algorithm. A real-time cloud-based data aggregation system, with connectivity to Cepheid's GeneXpert System *via* internet over data networks, was designed which permitted capture of real-time test specific data, including unique device identifier, geographic location, frequency of positive and negative results with running totals, by day, week, and month. The reporting relied on the FDA-cleared, Xpert Flu platform, which has high sensitivity and specificity for multiple influenza strains, a 1 h turn-around-time (TAT) and capacity for a cloud-based interface. Here, we assess clinician's perceived utility of this “prototype” molecular surveillance system.

We implemented a real-time influenza surveillance system that captured rapid molecular test results from two EDs. Here, we describe findings from a pre- and post-implementation survey of ED clinicians assessing their perceptions of this influenza system (electronic surveillance dashboard, and regular email updates generated from the dashboard). Findings may help guide approaches for collecting and distributing respiratory disease surveillance information with front-line ED clinicians.

## Materials and Methods

Patients presenting with an acute respiratory illness to The Johns Hopkins Hospital ED and Olive View-UCLA Medical Center ED were systematically tested for influenza using Xpert Flu (Cepheid, Sunnyvale, CA) from November 2013 to April 2014 (13). Both study sites are urban, academic EDs that provide health care to a highly underserved racial/ethnically minority patient population. Results of de-identified influenza test data from the GeneXpert platforms in both EDs were automatically uploaded to a real-time cloud-based data aggregation system, and immediately displayed on an electronic dashboard as cumulative daily tests performed and test result for influenza A, B, or H1N1 2009 viruses. Users also had options to display daily, weekly or seasonal trends. Given this was a prototype system, the dashboard was equipped with controlled access, appropriate security and privacy.

**Figure 1:**
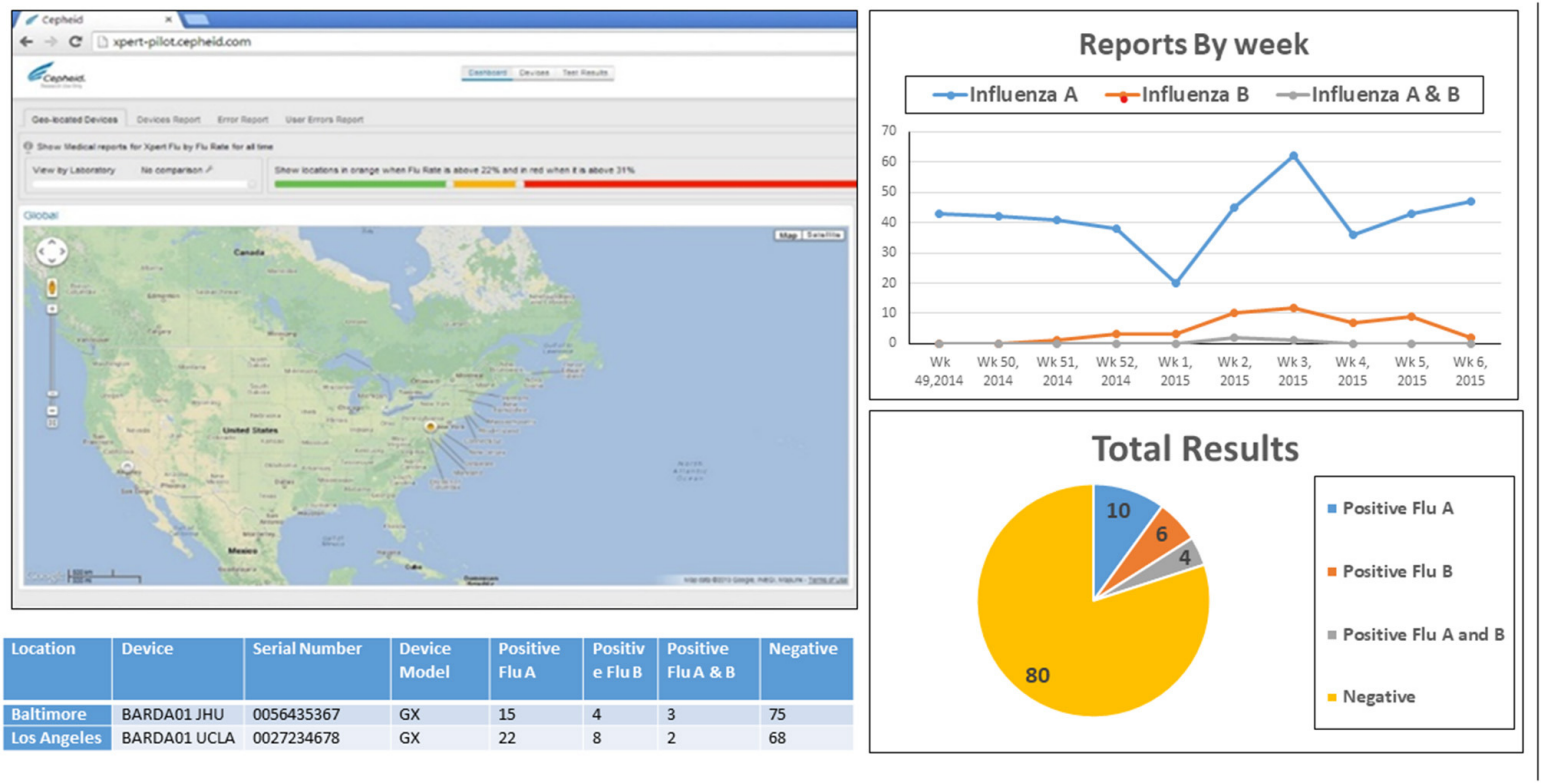
Webpage of the real-time, cloud-based and laboratory-based influenza surveillance system. Visual of Web accessible dashboard which displayed influenza testing data in real-time (as tests were performed) from the two emergency department testing sites. Results shown (below map) were real time view of daily test counts, and test results (positive or negative) for influenza A and influenza B. Display of results could be manipulated by users to show seasonal trend (top), total cumulative test distribution to date (bottom) for each site or both sites combined (based on viewer access privileges).

At the start of the influenza season, ED clinicians (residents, advanced practice providers and attending physicians) were briefed regarding the real-time prototype surveillance system, and given instructions for using the secure electronic dashboard (Figure 1) and the website address with secure login and password information. They were also informed that they would receive bi-monthly emails with summary reports of the aggregate ED testing data from the surveillance system.

ED clinicians were invited to participate in a baseline survey prior to implementation of the surveillance system and a follow-up survey after the influenza season. The anonymous survey included questions about demographics, years in practice, self-reported method(s) for obtaining information regarding influenza activity, and clinical practice patterns regarding testing, diagnosis, and antiviral treatment. The follow-up survey included additional questions pertaining to perceived utility and impact of surveillance information (both web-based dashboard and email communications) on testing, diagnosis, and antiviral treatment practices. Chi-square test or Fisher's exact test was performed to compare acceptability, perception, and clinical practice between baseline and follow-up. Difference in proportions and the corresponding 95% confidence interval (CI) was used to determine the association with an increase or decrease in a specific binary response between two time points. *P*-value was used to determine the difference in distribution of groups between surveys for a question with 3 or more mutually exclusive responses. *P*-value < 0.05 was considered as significant. Both study site Institutional Review Boards approved this study.

## Results

Overall, 151 (76%) of 198 ED clinicians participated in the baseline survey. Most were male (56%), <40 years old (70%), and physicians (91%), with 2-9 years of clinical practice (55%). Among 151 participants at the baseline, 87 (58%) responded to the follow-up survey. Demographics of participants who responded to the follow-up survey were similar to those at the baseline survey (Table 1).

**Table 1:** Characteristics of participants at the baseline and follow-up survey.

**Characteristics**	**Category**	**Baseline**	**Follow-up**	***P*-value**
		***n* = 151**	***n* = 87**	
Age group (years)	18-29	42 (28)	17 (20)	0.674
	30-39	64 (42)	40 (46)	
	40-49	25 (17)	19 (22)	
	50-59	13 (9)	7 (8)	
	≥60	6 (4)	4 (5)	
	Missing	1 (1)	0 (0)	
Sex	Female	63 (42)	43 (49)	0.205
	Male	84 (56)	44 (51)	
	Missing	4 (3)	0 (0)	
Position	Physician Resident	73 (48)	40 (46)	0.713
	Physician Assistant/ Nurse Practitioner	14 (9)	11 (13)	
	Attending Physician	64 (42)	36 (41)	
Years in Practice	0-1	26 (17)	13 (15)	0.971
	2-4	58 (38)	36 (41)	
	5-9	25 (17)	16 (18)	
	10-14	16 (11)	7 (8)	
	15-19	9 (6)	6 (7)	
	≥20	17 (11)	9 (10)	

Pre- and post-survey responses are summarized in Table 2. At baseline, 18% of clinicians reported that they regularly (weekly or monthly) “actively” obtained information about the influenza season and 51% “passively” obtaining information regularly.

**Table 2:** Survey responses regarding impact of influenza surveillance information on clinical practice before and after the implementation of an emergency department prototype real-time surveillance system.

**Questions**	**Categories**	**Baseline (%)**	**Follow-up (%)**	***P*-value or difference in proportion (95% CI)**
		***N* = 151**	***N* = 87**	
How often do you actively obtain information about the influenza season?	Several times a week	4 (3)	0 (0)	0.457
	Weekly	11 (7)	8 (9)	
	Monthly	12 (8)	5 (6)	
	Sporadically	92 (61)	51 (59)	
	I don't	32 (21)	23 (26)	
How often do you passively obtain information about the influenza season?	Several times a week	16 (11)	9 (10)	0.212
	Weekly	42 (28)	26 (30)	
	Monthly	18 (12)	20 (23)	
	Sporadically	70 (46)	31 (36)	
	I don't	4 (3)	1 (1)	
	Missing	1 (1)	0 (0)	
How do you track influenza prevalence? (select all that apply)	General news sources	60 (40)	36 (41)	2 (−10, 15)
	Emails from the ED/hospital regarding flu season	104 (69)	73 (84)	16 (4, 26)
	Weekly emails from hospital/clinical directors	39 (26)	19 (22)	−4 (−15, 7)
	I don't track	35 (23)	8 (9)	−14 (−23, −5)
	CDC website	32 (21)	19 (22)	1 (−10, 12)
	Other	3 (2)	3 (3)	1 (−3, 6)
Do you think additional information on influenza prevalence would be helpful to your clinical practice?	Yes	113 (75)	57 (66)	0.111
	No	38 (25)	29 (33)	
	Missing	0 (0)	1 (1)	
Which additional information on influenza prevalence would you find helpful to your clinical practice? (select all that apply)	Website geared to EM providers	55 (49)^*^	31 (54)^*^	6 (−10, 22)
	Emails updates on significant changes in epidemic	84 (74)^*^	45 (79)^*^	5 (−9, 18)
	Weekly email updates	27 (24)^*^	8 (14)^*^	−10 (−22, 2)
	Access to local surveillance information	42 (37)^*^	19 (33)^*^	−4 (−19, 11)
When do you test for influenza? (select all that apply)	All patients with respiratory illness in flu season	15 (10)	11 (13)	3 (−6, 11)
	Patients with ILI symptoms during season	33 (22)	25 (29)	7 (−5, 18)
	Only patients I clinically suspect have flu	44 (29)	23 (26)	−3 (−14, 9)
	Only patients I intend to treat with antivirals	41 (27)	27 (31)	4 (−8, 16)
	Only when required by my hospital guidelines	44 (29)	18 (21)	−8 (−20, 3)
	I don't test for influenza	11 (7)	5 (6)	−2 (−8, 5)
	Missing	4 (2)	2 (2)	Not performed
If I give an antiviral, I test for influenza	Almost always true	42 (28)	27 (31)	0.855
	Usually true	44 (29)	23 (26)	
	Occasionally true	32 (21)	22 (25)	
	Usually not true	16 (11)	8 (9)	
	Almost never true	12 (8)	6 (7)	
	I don't treat with antivirals	5 (3)	1 (1)	
	Missing	0 (0)	0 (0)	
I treat with an antiviral before I receive a positive influenza test	Almost always true	22 (15)	9 (10)	0.846
	Usually true	42 (28)	26 (30)	
	Occasionally true	45 (30)	28 (32)	
	Usually not true	15 (10)	11 (13)	
	Almost never true	16 (11)	11 (13)	
	I don't test for influenza	4 (3)	1 (1)	
	I don't treat with antivirals	5 (3)	1 (1)	
	Missing	2 (1)	0 (0)	
If you do treat with an antiviral prior to receiving a positive influenza test, when do you do so?(select all that apply)	I never treat before a positive test	6 (4)	3 (3)	0 (−5, 4)
	If the patient appears ill	96 (64)	66 (76)	12 (0, 24)
	If I am discharging the patient	28 (19)	20 (23)	4 (−6, 15)
	If I clinically suspect influenza	93 (62)	48 (55)	−6 (−19, 7)
	If my flu test takes several hours to result	35 (23)	26 (30)	7 (−5, 18)
	If I have a negative test, which has low sensitivity	22 (15)	13 (15)	0 (−9, 10)
	Other	15 (10)	5 (6)	−4 (−11, 3)
Please indicate the reasons you would NOT use an antiviral for a hospitalized patient with laboratory-confirmed influenza (select all that apply)	The patient presented >48 hours after symptom onset	103 (68)	54 (62)	−6 (−19, 6)
	Not sick enough to need antiviral treatment	30 (20)	21 (24)	4 (−7, 15)
	I don't believe antiviral medications work	3 (2)	4 (5)	3 (−2, 8)
	I don't want to promote antiviral resistance	9 (6)	3 (3)	−3 (−8, 3)
	Other	19 (13)	17 (20)	7 (−3, 17)

* *Percentages calculated based on the number of participants who responded “Yes” to the question “Do you think additional information on influenza prevalence would be helpful to your clinical practice?” The denominator was 113 for the baseline survey and that was 57 for the follow-up survey*.

Findings from the post-implementation survey were similar to those in the baseline with the following exceptions. First, there was an absolute increase (13%, 95% CI: 0%, 26%) in the proportion of clinicians who reported passively obtaining regularly information about influenza activity *at least* monthly (i.e., several times a week, weekly, or monthly) from 50 to 63%. Second, there was an absolute decrease in the proportion of clinicians who indicated they did not track influenza prevalence from 23 to 9% (difference: −14%, 95% CI: −23%, −5%) (Table 2). Clinicians reported an absolute increase (16%, 95% CI: 4%, 26%) in use of emails from the ED/hospital as the main information source to track influenza. There was also an absolute increase (12%, 95% CI: 0%, 24%) in the proportion of clinicians who indicated they prescribe antivirals to patients who appear ill prior to receiving a positive test.

Survey questions focusing on usefulness of the prototype surveillance dashboard and regular email updates (Table 3) revealed that 45% said that they went to the dashboard at least some time during the season (weekly 5%; monthly 2%; sporadically 38%). Among those reporting using the dashboard (*n* = 39), 78% indicated the dashboard increased their general awareness of influenza activity. A similar response pattern was seen regarding email communications from the prototype system, with 75% indicating that the email updates increased their general awareness of influenza.

**Table 3:** Survey responses regarding use and perceived usefulness of the prototype influenza surveillance system (dashboard and email updates) from 87 Emergency Department Clinicians post-influenza season.

**Question**	**Response**	**Number (%)**
How often did you go to the influenza surveillance website?	Several times a week	0 (0)
	Weekly	4 (5)
	Monthly	2 (2)
	Sporadically	33 (38)
	Never	45 (52)
	Don't Know	1 (1)
	Missing	2 (2)
How was the influenza surveillance website useful?	Increased my general awareness of influenza	31 (78)^*^
	Impacted my clinical diagnosis of influenza throughout the season	8 (20)^*^
	Impacted my decision-making to test for influenza	5 (13)^*^
	Impacted my decision making to treat for influenza	5 (13)^*^
	Impact on clinical diagnosis, testing, or treatment	10 (26)^*^
	Not useful	1 (3)^*^
	Don't Know	7 (18)^*^
How useful were the influenza surveillance emails?	Increased my general awareness of influenza	65 (75)
	Impacted my clinical diagnosis of influenza throughout the season	22 (25)
	Impacted my decision making to test for influenza	28 (32)
	Impacted my decision making to treat for influenza	20 (23)
	Impact on clinical diagnosis, testing, or treatment	42 (48)
	Not useful	6 (7)
How did having influenza surveillance data impact your	I tested more often	37 (43)
influenza testing practices?	I tested less often	2 (2)
	I chose to test based on the influenza prevalence	18 (21)
	The information did not impact my influenza testing practices	27 (31)
	Missing	3 (3)
How did having influenza surveillance data impact your	I gave more influenza antivirals	21 (24)
influenza treatment practices?	I gave less influenza antivirals	4 (5)
	I chose to treat based on the influenza prevalence	18 (21)
	The information did not impact my influenza treatment practices	41 (47)
	Not sure	1 (1)
	Missing	2 (2)

* *Denominator was the 39 individuals who reported using the influenza surveillance dashboard*.

Regarding perceived impact of having additional influenza surveillance data on the testing practices, 66% of participants indicated that having closer to real-time surveillance data impacted their clinical *testing* practice (including 43% testing more, 21% testing based on prevalence, and 2% testing less). Further, 50% indicated that having access to the surveillance data impacted their *treatment* practices (including 24% treating more often, 21% treating based on influenza prevalence, and 5% treating less often) (Table 3).

## Discussion

While influenza surveillance systems have potential usefulness for clinical settings, traditional methods for data sharing require gathering and collating data prior to data-sharing resulting in limited utility for front-line clinicians (11). Development and deployment of improved real-time data collection and reporting systems creates opportunities for sharing information more rapidly (6, 14, 15). Our survey of front-line providers before and after implementation on a prototype real-time cloud-based surveillance system suggests front-line clinicians perceive this as an added value for informing testing and treatment practices during a typical influenza season.

Very few published studies have assessed the potential utility of influenza surveillance for clinicians, and none specifically in the ED as far as we are aware. One study piloted various set triggers and real-time alerts for notifying physicians, finding that point-of-care testing and predefined alerts hold promise as tools for improving testing and/or treatment practices (16). Another systematic review found that local, real-time respiratory infectious disease surveillance data can increase antibiotic stewardship and antiviral prescribing practices (17). Our finding regarding the increased awareness and engagement associated with our influenza surveillance data sharing tools is important for ED clinicians, particularly given the clinical utility of prescribing appropriate antiviral to those at high risk for influenza complications. In addition, influenza surveillance system that includes near real-time influenza virus virulence data as well as antiviral resistance data would be able to further guide clinicians to modify their prescribing practice to optimize use of antivirals.

In our study, implementation of the dashboard and email updates was associated with an overall increase in clinician self-reported access to influenza surveillance data from 50 to 63%, and a decline in clinicians reporting never accessing influenza surveillance information. In addition, two-thirds and half of participants indicated that the added information impacted their overall testing and treatment practices, respectively. That effect observed from clinicians' self-reported responses was relatively small however, possibly due to clinician's uncertainty about how to use surveillance data to adjust their testing and treatment practices. In spite of being available in a dashboard web-based format, clinicians showed a preference for having updated surveillance information shared “passively” *via* email. This limits the true “real-time” value of the dashboard for situational awareness given that email updates were delivered only twice a month. Of note, the requirement for a separate secure login for clinicians to access the dashboard might have contributed to the relatively limited use of the dashboard.

Several limitations should be noted. First, the study occurred at two EDs; perceptions and uptake may not be generalizable to other settings. Second, although the characteristics of participants were similar between baseline and follow-up, more than 40% of participants did not return the follow-up survey. Those who responded to the follow-up survey are likely the ones who actually accessed the new surveillance platform. Therefore, the results of the follow-up survey are also partially reflective of the general attention and education provided as part of the implementation of the platform, rather than the use of the platform itself. Third, acceptability, perceptions, and self-reported clinical utility of the surveillance system is likely dependent on the magnitude and severity of each influenza season, and the 2013-2014 season was relatively mild. ED clinicians might find that the system has higher clinical utility in a more severe influenza season; stage of the influenza season (very early vs. later), may impact perceived utility, and that was not assessed here. Fourth, we measured perception, rather than actual impact on practice. Finally, newer testing and reporting systems have been advanced after this study was conducted, including those that are evolving in response to the global Coronavirus disease 2019 (COVID-19) pandemic (18). Findings from this front-line provider survey of a prototype system may be an informative baseline however, encouraging others to further advance and evaluate clinician's perceptions and practice patterns when interfacing with real-time reporting systems which take advantage of newer technologies and methods.

In conclusion, following implementation of a real-time reporting of influenza surveillance, ED clinicians reported increased awareness of influenza activity and modification of clinical practice patterns. Clinicians preferred receiving surveillance data passively *via* emails rather than proactively logging into a surveillance website. Further development of accurate and timely surveillance information distributed in easier to access formats for front-line ED clinicians may impact future management of patients with suspected influenza and other respiratory pathogens.

## Data Availability Statement

The raw data supporting the conclusions of this article will be made available by the authors, without undue reservation.

## Ethics Statement

The studies involving human participants were reviewed and approved by Johns Hopkins University School of Medicine and University of California Olive-View Medical Center. Written informed consent for participation was not required for this study in accordance with the national legislation and the institutional requirements.

## Author Contributions

RR and AFD contributed to conception and design of the study. AD, DT, GM, AK, and AFD had primary responsibility for the data collection. AD organized the database. Y-HH performed the statistical analysis. RR, Y-HH, DT, KS-S, and AFD primarily interpreted the results. Y-HH wrote the first draft of the manuscript. All authors contributed to manuscript revision, read, and approved the submitted version.

## Funding

The study was supported by US Department of Health and Human Services Biomedical Advanced Research and Development Authority (BARDA; agreement numbers IDSEP160031-01-00 and 130014-01-00). The funding bodies had no role in study design, data collection and analysis, preparation of the manuscript, or the decision to publish.

## Conflict of Interest

The authors declare that the research was conducted in the absence of any commercial or financial relationships that could be construed as a potential conflict of interest.

## Publisher's Note

All claims expressed in this article are solely those of the authors and do not necessarily represent those of their affiliated organizations, or those of the publisher, the editors and the reviewers. Any product that may be evaluated in this article, or claim that may be made by its manufacturer, is not guaranteed or endorsed by the publisher.
